# HER2-targeted antibody–drug conjugates for breast cancer: ancestry and dose adjustment for thrombocytopenia

**DOI:** 10.1007/s12282-023-01473-2

**Published:** 2023-06-16

**Authors:** Michael Rainone, Carolyn E. Behrendt, Saro Kasparian, Tina Nguyen, Mina S. Sedrak, Sayeh Lavasani, Daphne B. Stewart, Yuan Yuan, Joanne E. Mortimer, James R. Waisman, Niki Patel, Vinod Pullarkat

**Affiliations:** 1 Medical Oncology and Therapeutics Research, City of Hope Comprehensive Cancer Center, 1500 East Duarte Road, Duarte, CA 91101 USA; 2 Hematology and Hematopoietic Cell Transplantation, City of Hope Comprehensive Cancer Center, 1500 East Duarte Road, Duarte, CA 91101 USA; 3 Biostatistics, City of Hope Comprehensive Cancer Center, 1500 East Duarte Road, Duarte, CA 91101 USA; 4 Medical Oncology, Cedars-Sinai Medical Center, 127 South San Vicente Blvd, Los Angeles, CA 90048 USA

**Keywords:** HER2 positive breast cancer, Trastuzumab deruxtecan, Trastuzumab emtansine, Thrombocytopenia, Asian ancestry

## Abstract

**Background:**

Thrombocytopenia is a common adverse event on HER2-targeted therapies, fam-trastuzumab deruxtecan (T-DXd) and ado-trastuzumab emtansine (T-DM1). A reported association of Asian ancestry with this event merits investigation to rule out potential confounding.

**Methods:**

Subjects in this retrospective cohort were female patients with HER2 positive breast cancer, of Asian or non-Hispanic White ancestry, who initiated T-DM1 or T-DXd from January 2017 through October 2021. Follow-up closed in January 2022. Primary endpoint was dose adjustment for thrombocytopenia. Competing endpoints were discontinuation of drug for other toxicity, disease progression, or for completion of prescribed cycles. The association between Asian ancestry and thrombocytopenia-related dose adjustment was tested at *p* < 0.01 in a proportional hazards model for the sub-distributions of 4 (primary and competing) endpoints. Covariates examined as potential confounders were age, metastatic disease, specific HER2-targeted drug, and prior drug switching for toxicity.

**Results:**

Among 181 subjects, 48 reported Asian ancestry. Incidence of dose adjustment for thrombocytopenia was higher in patients with Asian ancestry and among patients switched to T-DXd after experiencing thrombocytopenia on T-DM1. Independent of specific drug and prior drug switching, Asian ancestry was associated with dose adjustment for thrombocytopenia (hazards ratio 2.95, 95% confidence interval 1.41–6.18) but not with competing endpoints. Among participants of Asian ancestry, the ancestral origin was usually China or the Philippines (where Chinese ancestry is common).

**Conclusions:**

The association between Asian ancestry and thrombocytopenia on HER2-targeted therapy is independent of age, metastatic disease, drug, and history of similar toxicity. This association may have a genetic basis linked to Chinese ancestry.

## Introduction

HER2-targeted antibody–drug conjugates approved for marketed use include fam-trastuzumab deruxtecan (T-DXd) and ado-trastuzumab emtansine (T-DM1). Among patients receiving either drug, thrombocytopenia is a frequently observed adverse event, as documented in the pivotal trials [1–5] that led to these drugs’ approval as treatments for metastatic HER2-positive breast cancer, metastatic HER2-low breast cancer [1, 4], and residual invasive HER2-positive breast cancer [3]. Since those regulatory approvals, T-DXd has been approved to treat other solid tumors, specifically HER2-mutant metastatic non-small cell lung cancer and HER2-positive advanced gastric or gastroesophageal junction adenocarcinoma [6].

Thrombocytopenia among patients taking T-DXd or T-DM1 is clinically significant, because it often results in dose-delay, dose-reduction, and discontinuation of these therapies [1, 3, 4, 7, 8]. Grade 3–4 thrombocytopenia has been reported in 5.7% [3], 12.9% [5], and 24.9% [4] of subjects on T-DM1 and 7.0% of subjects on T-DXd [4]. Thrombocytopenia has not been shown to have a relationship with poor prognosis; however, this is a concern when there is deviation from the protocol schedule due to dose-reductions or dose-delays, which have been shown to result in disease progression in other cancers [9]. Chemotherapy-induced thrombocytopenia has been shown to increase risk of serious bleeding events, hospitalization, and increased costs of healthcare [10]. Of note, this adverse reaction is not entirely random. According to meta-analysis of 25 studies of T-DM1 monotherapy [11], grade 3–4 thrombocytopenia is markedly associated with Asian ancestry, affecting 46% of patients with Asian ancestry only, 41% of those with mixed ancestry, but 19% of patients without Asian ancestry. The latter analysis was unable to control potential confounding of this association by age and other relevant factors.

To further investigate this association and consider potential confounding factors, we undertook a retrospective cohort study of breast cancer patients who received T-DXd or T-DM1 in the real-world setting of a tertiary referral cancer center. Our aim was to investigate whether Asian ancestry increases the risk of thrombocytopenia-related dose-adjustment of an HER2-targeted antibody–drug conjugate independently of age, metastatic disease, specific drug, and prior dose adjustment for toxicity. In addition, we describe the countries of ancestral origin within the designation of Asian ancestry, to inform future genetic study into the association between ancestry and this clinically important adverse drug reaction.

## Patients and methods

For this single-institution, retrospective study, a waiver of informed consent was granted by the medical center’s Institutional Review Board. Eligible for study were female patients with HER2 positive breast cancer who initiated T-DM1 or T-DXd from January 2017 through October 2021 and who at the time of their cancer diagnosis had self-reported their ancestry as either Asian or non-Hispanic White. To further define Asian ancestry, the likely country of ancestral origin was derived from the first and last names in the medical record.

For each patient, the drug(s) prescribed, their observed toxicity, and the number of drug cycles received were abstracted from the electronic medical record between November 2021 and January 2022. The study’s primary endpoint was dose adjustment of T-DM1 or T-DXd on account of thrombocytopenia. Individuals who experienced the primary endpoint on T-DM1 and then were switched to T-DXd contributed a second observation post-switch, thus were at risk of experiencing the primary endpoint a second time. The primary endpoint could be preempted by any of 3 competing endpoints: discontinuation of drug for toxicity other than thrombocytopenia, discontinuation of drug for disease progression, or completion of all prescribed cycles of drug. Absent a primary or competing endpoint, observation on study was censored at the close of study in January 2022.

The study’s pre-specified hypothesis was that breast cancer patients of Asian ancestry are at greater risk for a thrombocytopenia-related dose adjustment of T-DM1 or T-DXd than are patients without Asian ancestry (here, non-Hispanic Whites). This hypothesis was tested for statistical significance at *p* < 0.01 in a proportional hazards model for the sub-distributions of 4 endpoints (the primary endpoint and 3 competing endpoints) [12]. That model included a robust sandwich estimate to recognize correlation between repeated observations (before and after drug switching). Considered as potential covariates were age, metastatic disease, drug, and history of dose adjustment for thrombocytopenia or another toxicity. The final model for each endpoint retained those covariates that improved the model’s fit to the observed data, as indicated by a reduction in the Akaike Information Criterion.

## Results

Patients excluded per protocol were those who declined to self-identify ancestry (*n* = 23), self-identified as other than Asian or White (*n* = 28), and/or dissented to research (*n* = 24). The final cohort included 181 individuals (mean age 55.1 + 12.8 years), of whom 48 (26.5%) identified as Asian and 124 (68.5%) had metastatic disease at the start of follow-up. T-DXd was prescribed solely to patients with metastatic disease, while T-DM1 was prescribed in both the metastatic and the adjuvant (residual disease) setting. All patients had received prior chemotherapy with a taxane and trastuzumab. Thirty-three patients received T-DXd without having received prior T-DM1, the remainder received prior T-DM1, a majority of which received it as a 3rd line treatment following therapy with T-DM1 in the 2nd line setting. Baseline platelet counts are shown in Table 1. Overall, 33 individuals received T-DXd exclusively, and another 45 were switched to that drug after developing thrombocytopenia on T-DM1.

**Table 1:** Baseline characteristics

	White (*N* = 133)	Asian (*N* = 48)
Age 20–39	11 (8%)	3 (6%)
Age 40–59	63 (47%)	29 (60%)
Age 60–79	53 (40%)	13 (27%)
Age 80+	6 (5%)	3 (6%)
Metastatic disease	94 (71%)	30 (62%)
Bone marrow^a^	2	0
Liver	30	7
Brain	33	15
Bone	74	15
Localized (non-metastatic)	39 (29%)	18 (38%)
Estrogen or progesterone receptor positive	96 (72%)	33 (69%)
Platelets/µL^b^		
70–149	12 (9%)	1 (2%)
150–200	26 (20%)	15 (31%)
201–300	64 (49%)	29 (60%)
301–449	25 (19%)	3 (6%)
450–667	5 (4%)	0 (0%)

^a^Assessed by PET CT

^b^Platelet count prior to initiation of HER2 antibody–drug conjugate therapy, by ancestry; note pre-treatment platelet count was unavailable for 1 white subject

Subjects contributed 181 observations pre-switch and another 45 post-switch. These 226 observations included a total of 2551 cycles of treatment with T-DM1 or T-DXd. Observations ended in either dose adjustment for thrombocytopenia (*n* = 32), discontinuation of drug for toxicity other than thrombocytopenia (*n* = 64), discontinuation of drug for disease progression (*n* = 48, all with metastatic disease at baseline), completion of treatment (*n* = 27, none with metastatic disease at baseline), or close of study (*n* = 55).

**Fig. 1:**
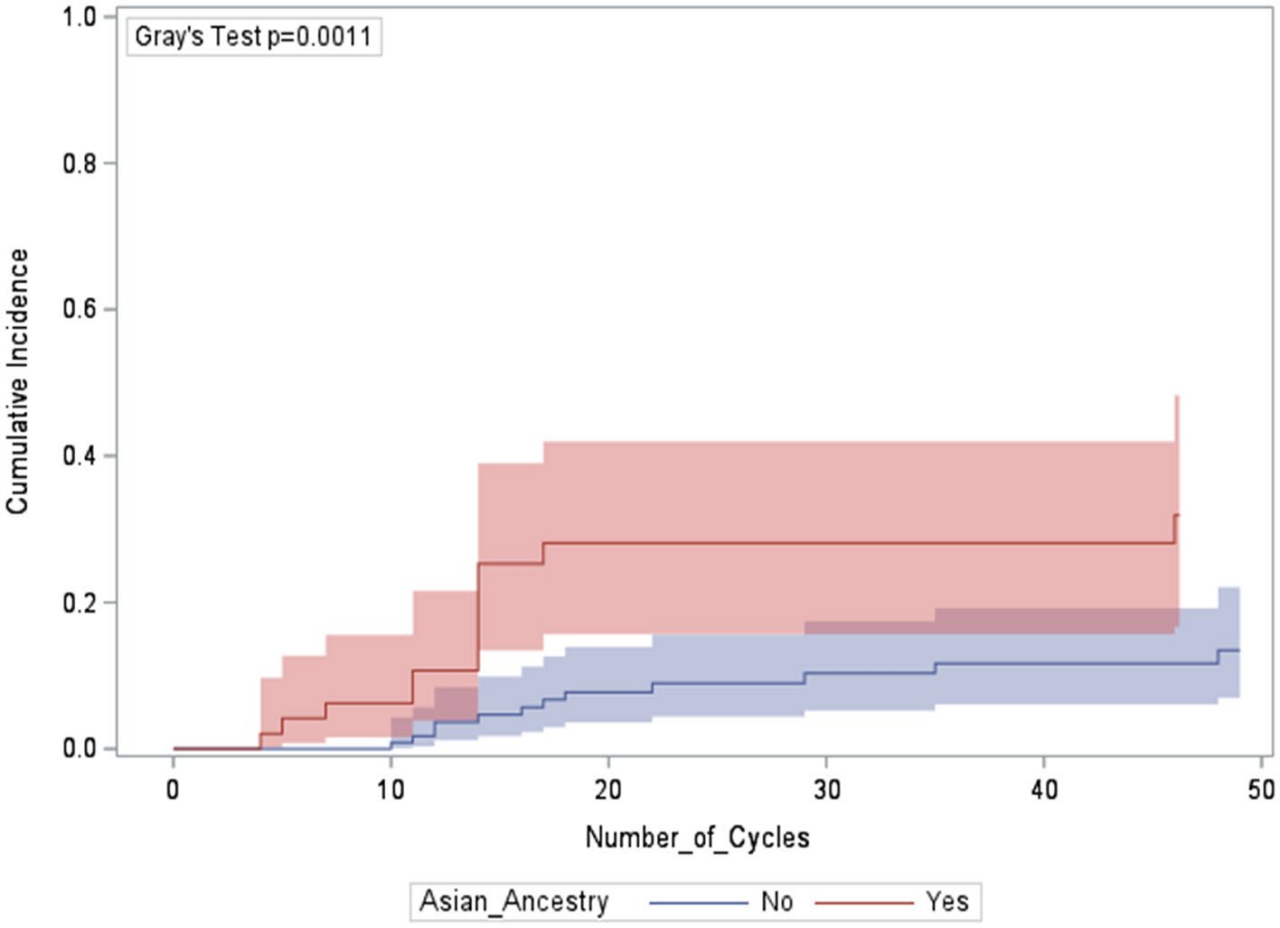
Cumulative incidence of a first dose adjustment for thrombocytopenia, by ancestry (*N* = 181)

As shown in Fig. 1, Asian ancestry accelerated time to first dose adjustment for thrombocytopenia. On univariable analysis (Table 2), the incidence of dose adjustment for thrombocytopenia per 100 patient-cycles of drug was elevated in 2 subgroups: patients with Asian ancestry and patients who were switched to T-DXd after experiencing thrombocytopenia on T-DM1. Of the *n* = 181 unique subjects, 4 (including 3 with Asian ancestry) experienced the primary endpoint twice, first on T-DM1, then on T-DXd. As shown, the incidence of dose adjustment for thrombocytopenia did not vary by age or stage of disease.

**Table 2:** Incidence of dose adjustment for thrombocytopenia, by patient characteristics

	*N* subjects	*N* cycles	*N* events^a^	Events per 100 cycles	95% confidence interval	Unadjusted *p*^†^
Asian ancestry						0.0021
Yes	48	643	16	2.49	(1.52–4.06)	
No	133	1908	16	0.84	(0.51–1.37)	
Stage of tumor						0.4919
Metastatic	124	1890	22	1.16	(0.77–1.77)	
Residual non-metastatic	57	661	10	1.51	(0.81–2.81)	
Age						0.8192
27–54 years	90	1327	16	1.21	(0.74–1.97)	
55–87 years	91	1224	16	1.31	(0.80–2.13)	
Drug, by treatment history						
T-DM1, as initial drug	148	1749	24	1.37	(0.92–2.05)	^‡^
T-DXd, as initial drug	33	306	1	0.33	(0.05–2.32)	0.1598
T-DXd, after stopping T-DM1						
For thrombocytopenia	9	67	4	5.97	(2.24–15.91)	0.0065
For other toxicity	36	429	3	0.70	(0.23–2.17)	0.2710

^†^In this stage of the current analysis, *p* values are not adjusted for covariates, competing risks, or multiple hypothesis testing; ^‡^T-DM1 is the referent category for comparing event incidence across the categories of exposure to drug

^a^Event refers to dose adjustment for thrombocytopenia

On multivariable analysis, Asian ancestry was significantly associated with the primary endpoint (dose adjustment for thrombocytopenia) independent of drug and history of drug switching (Table 3).

**Table 3:** Proportional hazards model of the primary and competing endpoints

	Dose adjustment for thrombocytopenia	Discontinuation for other toxicity	Discontinuation for progression	Completion of prescribed cycles
	Hazards ratio (95% confidence interval) of the sub-distribution of a competing risk			
Asian ancestry				
Yes	2.95 (1.41–6.18)*	1.14 (0.67–1.95)	0.58 (0.23–1.46)	0.68 (0.32–1.43)
No	1.00	1.00	1.00	1.00
Drug, by treatment history				^a^
T-DM1, as initial drug	1.00	1.00	1.00	
T-DXd, as initial drug	0.21 (0.03–1.42)	0.05 (0.01–0.36)	5.28 (2.66–10.47)	
T-DXd, after stopping T-DM1				
For thrombocytopenia	5.96 (2.38–14.94)	0	0	
For other toxicity	0.89 (0.27–2.94)	0.19 (0.05–0.73)	4.18 (2.54–6.86)	
Per 100 unit increase in age^2^	^a^	1.02 (1.00–1.03)	^a^	0.975 (0.951–0.999)
Stage of disease	^a^		^b^	^b^
Metastatic Residual non-metastatic		3.06 (1.54–6.07) 1.00		

*Significant association, *p* = 0.004. In the 4 sub-models, statistical significance was evaluated solely for the hypothesized risk factor, Asian Ancestry

^a^Along with Asian Ancestry, each sub-model included only those covariates that improved its fit to the observed data

^b^Discontinuation for disease progression occurred only among patients being treated for metastatic disease, while completion of prescribed cycles occurred only among patients being treated for residual non-metastatic disease

In contrast to its association with the primary endpoint, Asian ancestry was not associated with any competing endpoints. Instead, those endpoints varied by drug, age, and metastatic disease (Table 3).

For 43 of the 48 subjects who reported Asian ancestry, first name and/or surname suggested a country of ancestral origin. That country was most often China [*n* = 23, including 9 patients who developed thrombocytopenia once (*n* = 6) or twice (*n* = 3)] or the Philippines (*n* = 12, including 2 patients who developed thrombocytopenia once). The remaining Asian-identifying patients included *n* = 8 with names suggestive of Burmese, Indonesian, Indian, Japanese or Korean ancestry (none of whom developed thrombocytopenia) and *n* = 5 patients who could not be assigned a country of ancestral origin (including 2 patients who developed thrombocytopenia once).

## Discussion

The current study confirms that, among women taking T-DM1 and/or T-DXd for HER2 positive breast cancer, Asian ancestry is associated with greater risk of dose adjustment for thrombocytopenia in a real-world setting. As we show, this risk is specific to thrombocytopenia and is unrelated to other toxicity or disease progression. Our analysis also demonstrates that the association with Asian ancestry is not confounded by age, metastatic disease status, or recent history of thrombocytopenia on a similar drug. A limitation of our study is that potential confounding by body weight or body mass index could not be ruled out. Such potential confounding should be investigated in future studies of this adverse drug reaction. Furthermore, the individuals included in this study received prior cytotoxic chemotherapy, as taxanes form the backbone of neoadjuvant and first line metastatic therapy, which can potentially cause myelosuppression.

Among reports about thrombocytopenia on T-DM1 or T-DXd [11], the current study is the first to characterize patients with Asian ancestry according to their likely country of ancestral origin. However, the association of thrombocytopenia with Asian ancestry in individuals treated with T-DM1 has previously been characterized by Zhang et al*.* who showed in a meta-analysis that the incidence of grade ≥ 3 thrombocytopenia was 0.20 (95% CI 0.10–0.29), compared to 0.02 (95% CI 0.01–0.03) in non-Asian individuals treated with T-DM1 [11]. To our knowledge this is the first study to highlight this adverse event in individuals of Asian origin treated with T-DXd. Findings from our admittedly small sample suggest that a genetic basis for this association may be linked to Chinese ancestry, which is also common among the Filipino population. We hope that current findings will assist future investigations into the mechanism underlying this adverse drug reaction and may also inform clinical guidelines for its prevention and management.

The precise mechanism of thrombocytopenia secondary to these antibody–drug conjugates remains unknown. Studies have explored potential mechanisms of thrombocytopenia in T-DM1 use have found that there is likely a selective toxicity to megakaryocytes; however, not directly to the hematopoietic stem cells as it thought to be the mechanism in conventional chemotherapy [11, 13]. The mechanism of thrombocytopenia secondary to T-DXd remains unknown. This warrants further investigation as well as investigation into interventions to overcome this toxicity and maintain dose-intensity. The role of thrombopoietic receptor agonists as a rescue for chemotherapy-induced thrombocytopenia has been studied; however, not explicitly in the setting of HER2-targeted antibody–drug conjugates [14]. This intervention that warrants further investigation in clinical trials and has potential to be of benefit to both Asian populations, including those of Chinese origin, that are at a higher risk of this complication, and non-Asians that develop this adverse event leading to dose-reductions and therapy delays.

## Data Availability

Original data can be made available upon request of the corresponding author.
